# Evolutionary loss of thermal acclimation accompanied by periodic monocarpic mass flowering in *Strobilanthes flexicaulis*

**DOI:** 10.1038/s41598-021-93833-1

**Published:** 2021-07-12

**Authors:** Atsushi Ishida, Tomomi Nakamura, Shin-Taro Saiki, Jin Yoshimura, Satoshi Kakishima

**Affiliations:** 1 Center for Ecological Research, Kyoto University, Otsu, Shiga 520-2113 Japan; 2 Forestry and Forest Products Research Institute, Tsukuba, Ibaraki 305-8687 Japan; 3 Institute of Tropical Medicine, Nagasaki University, Sakamoto, Nagasaki, Nagasaki 852-8523 Japan; 4 Faculty of Science, Tokyo Metropolitan University, Minami-Osawa, Hachioji, Tokyo 192-0397 Japan; 5 The University Museum, The University of Tokyo, Hongo, Bunkyo, Tokyo 113-0033 Japan; 6 Center for Molecular Biodiversity Research, National Museum of Nature and Sciences, Tsukuba, Ibaraki 305-0005 Japan

**Keywords:** Ecology, Physiology, Plant sciences

## Abstract

While life history, physiology and molecular phylogeny in plants have been widely studied, understanding how physiology changes with the evolution of life history change remains largely unknown. In two closely related understory *Strobilanthes* plants, the molecular phylogeny has previously shown that the monocarpic 6-year masting *S. flexicaulis* have evolved from a polycarpic perennial, represented by the basal clade *S. tashiroi*. The polycarpic *S. tashiroi* exhibited seasonal thermal acclimation with increased leaf respiratory and photosynthetic metabolism in winter, whereas the monocarpic *S. flexicaulis* showed no thermal acclimation. The monocarpic *S. flexicaulis* required rapid height growth after germination under high intraspecific competition, and the respiration and N allocation were biased toward nonphotosynthetic tissues. By contrast, in the long-lived polycarpic *S. tashiroi*, these allocations were biased toward photosynthetic tissues. The life-history differences between the monocarpic *S. flexicaulis* and the polycarpic *S. tashiroi* are represented by the “height growth” and “assimilation” paradigms, respectively, which are controlled by different patterns of respiration and nitrogen regulation in leaves. The obtained data indicate that the monocarpic *S. flexicaulis* with the evolutionary loss of thermal acclimation may exhibit increased vulnerability to global warming.

## Introduction

Atmospheric carbon dioxide (CO_2_) concentrations are increasing, because of extensive fossil fuel burning. Climate changes predicted for the next century include a continued warming of air temperatures on the order of 2–7 °C^1^, and the increased air temperatures will be able to change carbon uptake rates of plants and to increase plant respiration rates^2,3^. Although the increase in air temperature is significant globally, the rate of air temperature increase varies regionally. Currently, the average air temperature is increasing by 0.85 °C per year in globally^4^, but the increase in Japan reaches 1.15 °C per year (according to the Japan Meteorological Agency). Leaf gas exchange of vascular plants plays an important role in regulating global carbon cycling^5,6^. To avoid thermal damage and to ensure carbon assimilation under seasonal variations in air temperature, terrestrial vascular plants usually adjust their photosynthesis and respiration to altered air temperatures^5–10^. Even tropical plants and C_4_ plants from warm habitats exhibit photosynthetic thermal acclimation to altered air temperatures, i.e., the net photosynthetic rates measured at a given temperature can be altered between plants growing under different air temperatures^11,12^. However, if the increasing air temperature exceed the optimum temperature for daily carbon gain in the whole plants beyond the range of thermal acclimation in cell metabolisms, such changes are projected to threaten survival of the plants. Such failure in thermal acclimation leads to local extinctions and range migrations of the plant population, and resultantly to altered forest composition and function^1,3,13^. To improve the prediction of the effects of plant thermal acclimation on terrestrial carbon cycling under global warming, numerous experiments have been conducted under regulated environments over short to long periods, and these results have been fed into meta-analyses across various functional types and biomes^6,10,14–17^.

The range of seasonal variations in air temperature exceeds 20 °C in the warm-temperate forests of southern Japan. The values of Q_10_ values (proportional change in respiration rates per 10 °C change in temperature) for the respiration rates of many vascular plants are around 2, meaning that the respiration rates increase two-fold with a 10 °C increase in temperature. The reported seasonal variation in Q_10_ has not been large or has been nonsignificant for various plant species^6,18,19^. Species-specific thermal acclimation of cell metabolism, which is measured at a given temperature over seasons, could be thus considered a physiologically important determinant of the vulnerability of plant growth and survival under global warming. The temperature response of mitochondrial respiration rates reflects metabolic activity of plant tissues^20,21^. Nevertheless, the information on thermal acclimation in rare and/or understory plants is virtually missing. Not only thermal acclimation^1–3^ but also the migration and colonization rates^13^ are the major determinant factors for predicting how native species will distribute themselves in response to habitat loss and global warming. Rare understory plants usually have low spread rates and low long-distance migration capacity^13^. Therefore, thermal acclimation may be the most important factor for the avoidance of extinction under global warming. However, because of the lack of knowledge in thermal acclimation capacity for these plants, we cannot evaluate the predicted rapid loss of rare species and resultant biodiversity under global warming.

**Figure 1:**
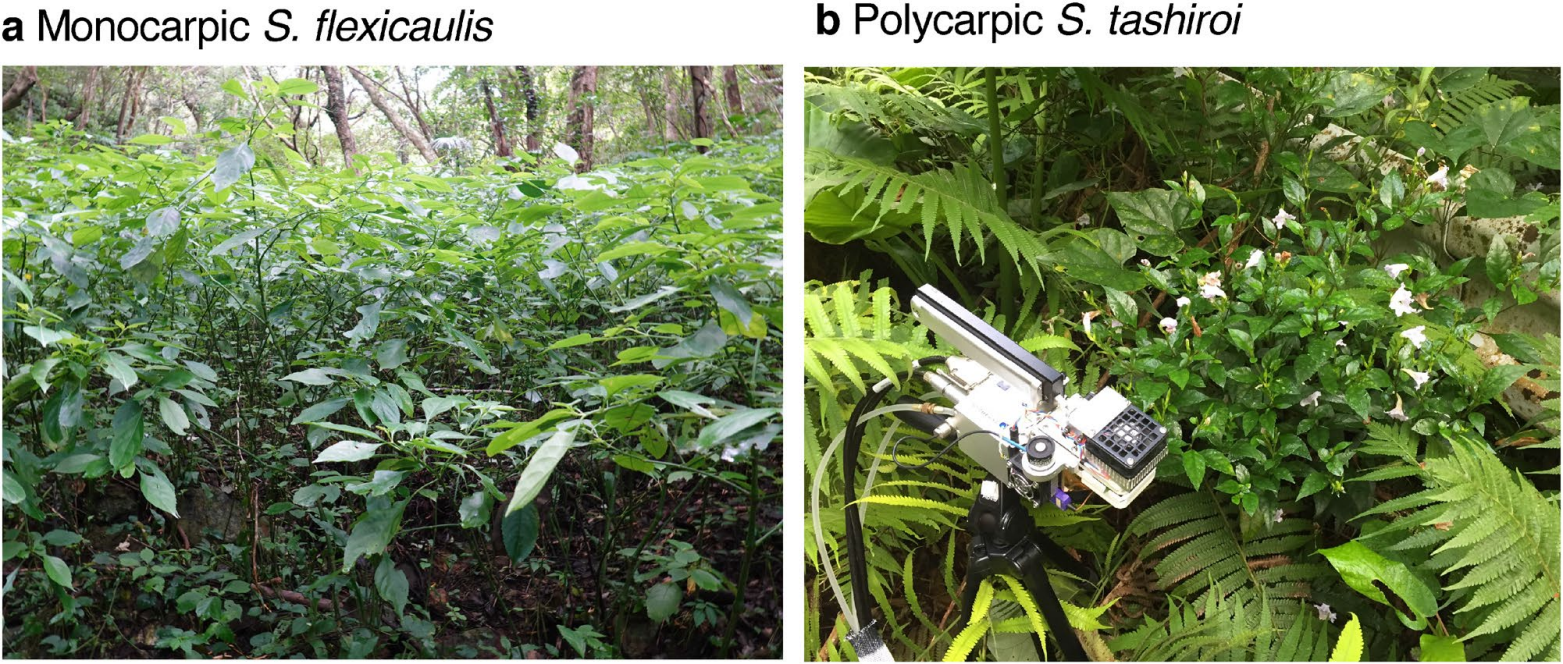
Two *Strobilanthes* species growing in the understory of evergreen forests in Japan. (**a**) Photographs of the monocarpic *S. flexicaulis*. (**b**) The polycarpic *S. tashiroi* with the chamber head of a portable open gas exchange system (LI-6400). The maximum top-canopy heights are approximately 2 m and 0.5 m high above the ground in *S. flexicaulis* and *S. tashiroi*, respectively*.* When the germination started in *S. flexicaulis* in spring 2016*,* the number of seedlings often exceeded 780 m^−2^ under the parent plants.

We examined two closely-related sympatric understory plants of the genus *Strobilanthes* species (Acathaceae), *S. tashiroi* Hayata and *S. flexicaulis* Hayata, in warm-temperate, overwintering evergreen forests in Japan (Fig. 1). They have contrasting life histories: *S. tashiroi* is a perennial herb with continuous flowering and non-masting behavior, whereas *S. flexicaulis* is a shrub with periodic monocarpic mass flowering and subsequent death every 6 years^22^. Molecular phylogeny shows that the periodical behavior of *S. flexicaulis* has locally evolved from the polycarpic behavior of *S. tashiroi*^23^.

Thermal acclimation of cell metabolism is expected to be different between the two plants with distinctive life histories (polycarpic vs. periodic monocarpic). Costly thermal acclimation may be disadvantageous for the monocarpic *S. flexicaulis*, which must undergo rapid height growth during its short lifespan, whereas *S. tashiroi*, thermal acclimation should be advantageous to ensure the effective photosynthesis over its long lifespan. Here, we examined the thermal acclimation and its related physiological states of the two *Strobilanthes* species in the field. The photosynthetic and dark respiration rates in leaves, stems, and roots were compared between summer and winter on Okinawa Island. We found that the monocarpic *S. flexicaulis* leaves did not show any seasonal acclimation in respiration and photosynthesis, whereas the polycarpic *S. tashiroi* leaves showed increased respiratory and photosynthetic faculties in winter. We discuss the loss of thermal acclimation in *S. flexicaulis* and its implications in the context of global warming causing the extinction of rare species and loss of biodiversity. This study is the first report that integrates physiological ecology (thermal acclimation) and plant life histories (polycarpic vs. periodic monocarpic) with molecular phylogeny. Until now, there has been no study linking thermal acclimation with the evolution of life history change in plant species.

## Results

### Seasonal variations in air temperature in the study site

From 2017 to 2020, the yearly maximum, minimum, and average air temperatures were 29.2 ± 0.49 °C, 5.8 ± 0.99 °C, and 19.3 ± 0.23 °C (mean ± 1 SD), respectively. No conspicuous variations in the seasonal trends were found among the past 4 years (Supplementary Fig. 1). The difference between the annual maximum and minimum air temperatures was 23.4 ± 0.69 °C (mean ± 1 S.D.) from 2017 to 2020. This large difference indicates a considerable seasonal variation in air temperature even in the forest understory. The observed results are typical climatological characteristics in the warm-temperate, evergreen forests in East Asia. There were no conspicuous drought and disturbance events, such as tropical typhoons, since 2016, when their gemination of *S. flexicaulis* began.

### Leaf area-based photosynthesis increases in winter in the polycarpic *S. tashiroi*

**Figure 2:**
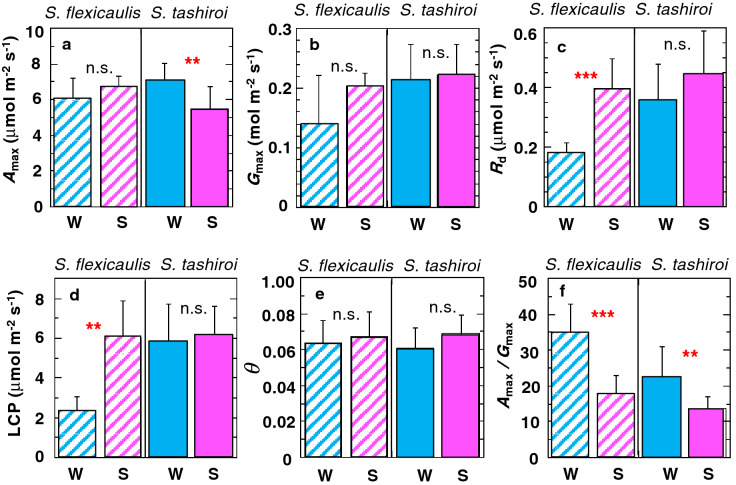
Leaf area-based gas exchange traits measured in field settings in the monocarpic *Strobilanthes flexicaulis* and the polycarpic *S. tashiroi* in winter (blue) and summer (pink)*.* (**a**) *A*_max_: maximum net photosynthetic rate. (**b**) *G*_max_: max. water–vapor stomatal conductance. (**c**) *R*_d_: dark respiration rate. (**d**) LCP: light compensation point. (**e**) *Φ*: initial slope of the light-response curve. (**f**) *A*_max_*/G*_max_ ratio. W and S show the winter and summer measurements, respectively. Bars show + 1 S.D. Significant differences were compared between seasons in each species (***P < 0.001, **P < 0.01, n.s.: *P* > 0.05).

The light-response curves of the photosynthesis rates were measured in winter and summer under nonregulated air temperature conditions in a field setting (Supplementary Fig. 2). The measured leaf temperature was 20–24 °C in winter, whereas it was 28–29 °C in summer. The two species exhibited different seasonal variations in leaf gas exchange (Fig. 2). In the monocarpic *S. flexicaulis,* the maximum net assimilation rates (*A*_max_) did not show seasonal variations (Fig. 2a), but dark respiration rates (*R*_d_) decreased significantly in winter (Fig. 2c). In contrast, in the polycarpic *S. tashiroi*, *A*_max_ increased significantly in winter (Fig. 2a), but *R*_d_ did not vary even though the winter temperatures were low (Fig. 2c). The *A*_max_/*R*_d_ ratio was significantly higher in summer than in winter in both species. However, the summer-to-winter ratios in *A*_max_/*R*_d_ were 1.9 and 1.6 in *S. flexicaulis* and *S. tashiroi*, respectively. The low summer-to-winter ratio in the polycarpic *S. tashiroi* indicates that they maintain a more homeostatic carbon balance over seasons with altered air temperatures (see^6^) than the monocarpic *S. flexicaulis. S. tashiroi* did not show the seasonal variations in light compensation points (LCPs), whereas the LCPs in *S. flexicaulis* decreased significantly in winter (Fig. 2d), because of low dark respiration rates in winter (Fig. 2c). The initial slopes of the light-response curves were kept at relatively high values (> 0.061) over the seasons in both species (Fig. 2e), indicating that the leaves did not suffer from conspicuous photoinhibition^24–26^.

### Cell metabolisms increases in winter in the polycarpic *S. tashiroi,* but not in the monocarpic *S. flexicaulis*

**Figure 3:**
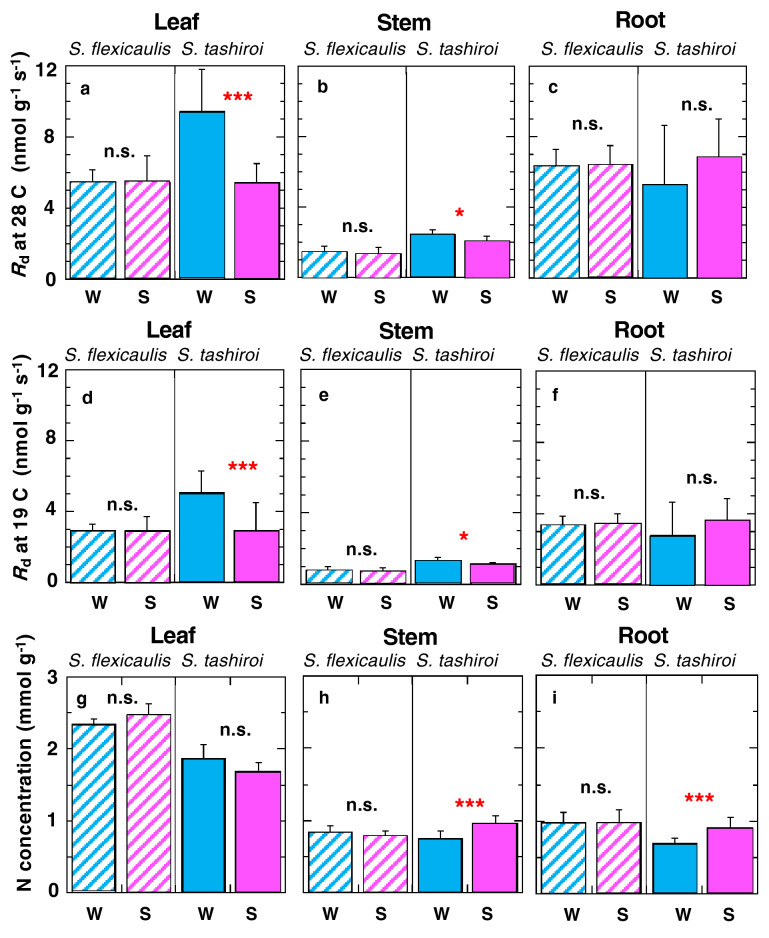
Dry mass-based dark respiration rates and N concentrations in each tissue in the monocarpic *Strobilanthes flexicaulis* and the polycarpic *S. tashiroi*. The respiration rates were measured in the field in each season, and the obtained values were standardized to a constant temperature (19 °C and 28 °C) by using Q_10_ = 2. (**a**, **d**, **g**) show data from leaves, (**b**, **e**, **h**) show data from stems, and (**c**, **f**, **i**) show data from roots. (**a**–**c**) Data at 28 °C, and (**d**–**f**) data at 19 °C. W and S show the winter and summer measurements, respectively. Bars show + 1 S.D. Significant differences were compared between seasons in each species (***P < 0.001, *P < 0.05, n.s.: *P* > 0.05).

**Figure 4:**
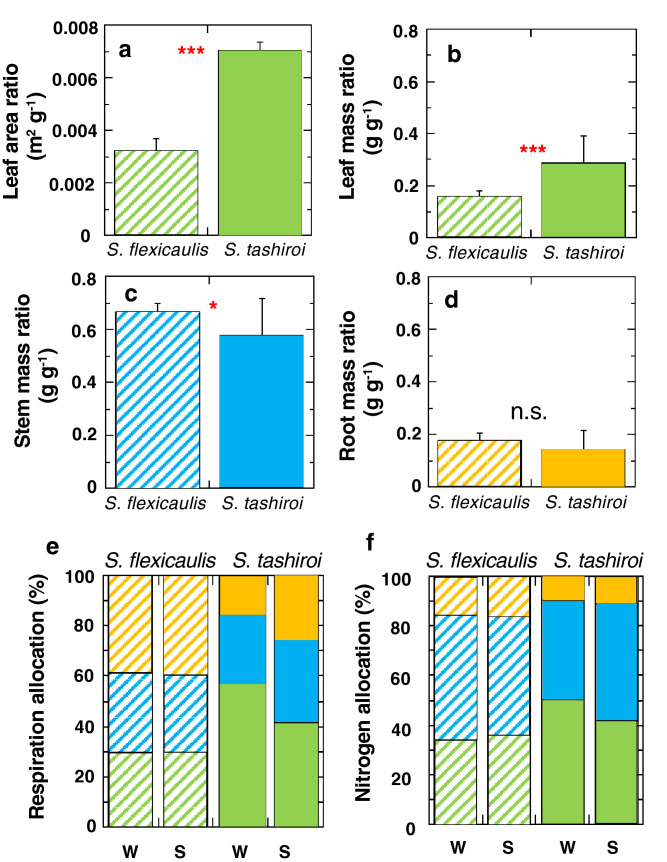
The allocation of dry matter, whole-plant respiration rates and nitrogen contents among tissues in the monocarpic *Strobilanthes flexicaulis* and the polycarpic *S. tashiroi*. (**a**) Leaf area ratio, (**b**) leaf mass ratio, (**c**) stem mass ratio, and (**d**) root mass ratio in plant dry matter. Bars show + 1 S.D. Significant differences in dry matter allocation were compared between species (***P < 0.001, *P < 0.05, n.s.: *P* > 0.05). (**e**) Dark respiration and (**f**) nitrogen allocations. W and S show the winter and summer measurements, respectively. Green: leaves; blue: stems; yellow: roots.

Mass-based dark respiration rates in leaves, stems, and roots were measured in winter and summer in the field. To standardize the variations in measurement temperature and to evaluate the seasonal variations in cell metabolism between summer and winter, the obtained values were standardized at 19 °C and 28 °C by using Q_10_ = 2 (Fig. 4a–f); these temperatures corresponded to the mean values of plant temperature measured in winter and summer in the field setting, respectively. The two species exhibited seasonally different responses in the respiration rates, especially in the aboveground tissues. Comparing between winter and summer, the respiration rates at a given temperature (i.e., metabolic activity) increased significantly in winter for the leaves and stems of polycarpic *S. tashiroi* (Fig. 3a,b,d,e). The respiratory metabolic activity of *S. tashiroi* leaves increased 1.7 times from summer to winter; this phenomenon corresponds to the increase in *A*_max_ observed in winter. In contrast, in the monocarpic *S. flexicaulis*, no metabolic change over seasons was found for any tissues. For the roots, no seasonal metabolic change in cell respiration was found in either species (Fig. 3c,f). Comparing between the two species, no significant differences in the mass-based root respiration rates were found, whereas the mass-based leaf and stem respiration rates were significantly lower in the monocarpic *S. flexicaulis* than in the polycarpic *S. tashiroi* (*P* = 0.001 for leaves; *P* < 0.001 for stems) in both seasons (Supplementary Table 2).

Similar to cell metabolisms, no seasonal variations in nitrogen (N) concentrations were found in any tissues in the monocarpic *S. flexicaulis* (Fig. 3g–i). In the polycarpic *S. tashiroi*, no seasonal variations in N concentrations in leaves were found (Fig. 3g), but those in stems and roots increased significantly in summer (Fig. 3h,i). Therefore, the seasonal variations in N concentrations were not consistent with those in the respiratory metabolism of *S. tashiroi* cells.

Photosynthetic N use efficiency (PNUE; N-based photosynthetic rate) was estimated from the mean values of *A*_max_ and foliar N. The values of PNUE in the polycarpic *S. tashiroi* were 0.191 and 0.147 μmol mmol N^−1^ in winter and summer, respectively; i.e., PNUE increased 1.3 times from summer to winter because *A*_max_ increased in winter, but foliar N remained unchanged. In the monocarpic *S. flexicaulis*, the PNUE values were 0.126 and 0.139 μmol mmol N^−1^ in winter and summer, respectively; i.e., PNUE did not vary largely between seasons. These data indicate that the N within the lamina is allocated mostly to photosynthetic enzymes (such as Rubisco) in winter in the polycarpic *S. tashiroi* leaves*,* whereas the allocation of N within the lamina did not vary largely in the monocarpic *S. flexicaulis* leaves*.* Thus, Rubisco abundance rather than N concentrations within the lamina underpins the thermal acclimation response in polycarpic *S. tashiroi* leaves*.*

### The respiration, N, and dry matter allocations vary between the two species

The two species have contrasting plant forms in relation to the differences in their life histories (Fig. 4a–d). The monocarpic *S. flexicaulis* had a significantly higher stem mass ratio than did the polycarpic *S. tashiroi* (Fig. 4c), whereas *S. tashiroi* had a significantly higher leaf mass ratio (resulting in a high leaf area ratio) than did *S. flexicaulis* (Fig. 4b). The high dry matter allocation into stems in the monocarpic *S. flexicaulis* is due to its unique life history, which requires rapid height growth during a short period (6 years) after germination to escape from high intraspecific competition (i.e., shading by the other individual plants). No significant difference between species was found in root dry mass (Fig. 4d). The values of leaf mass per area (LMA) were 48.3 ± 2.4 and 37.2 ± 4.5 g m^−2^ (mean ± 1 S.D.) in *S. flexicaulis* and *S. tashiroi*, respectively; LMA was significantly higher in *S. flexicaulis* than in *S. tashiroi* (*P* < 0.001) (Fig. 4a).

The whole-plant respiration rates in monocarpic *S. flexicaulis* were 1.57 and 2.92 nmol g^−1^ s^−1^ in winter (19 °C) and summer (28 °C), respectively, and those in polycarpic *S. tashiroi* were 2.59 and 3.70 nmol g^−1^ s^−1^ in winter and summer, respectively. In terms of interspecific variation, the whole-plant respiration rates in *S. tashiroi* were 1.7–1.3 times of those in *S. flexicaulis*, because the polycarpic *S. tashiroi* was leafier. In terms of seasonal variation, the summer-to-winter ratios of the whole-plant respiration rates were 1.86 and 1.43 in *S. flexicaulis* and *S. tashiroi*, respectively. Therefore, with regard to whole-plant respiration, the polycarpic *S. tashiroi* maintained higher homeostasis over seasons through the thermal acclimation of leaf respiration (see^21^) than did the monocarpic *S. flexicaulis*.

Interspecific differences were also found in respiration and N allocation among tissues (Fig. 4e,f). In the polycarpic *S. tashiroi*, leaf respiration amounted to approximately 42–55% of the whole-plant respiration rates (Fig. 4e), and leaf N amounted to approximately 42–50% of the whole N contents (Fig. 4f). On the other hand, in the monocarpic *S. flexicaulis*, leaf respiration amounted to approximately 29% of the whole-plant respiration rates (Fig. 4e), and leaf N amounted to approximately 34–36% of the whole N contents (Fig. 4f).

## Discussion

This is the first paper combing physiological traits and life histories (polycarpic vs. polycarpic) in plants. Until now, the evolutional process of life history is not directly compared with physiological acclimation capacity, because the life history differences are represented in distant phylogeny and direct comparisons are impossible. Furthermore, we cannot deeply know what physiological traits are correlated to the evolution of life history change. However, in this study, we examined two closely related plants with distinct life histories, and elucidated the ecophysiological differences that have supported their own life history.

**Figure 5:**
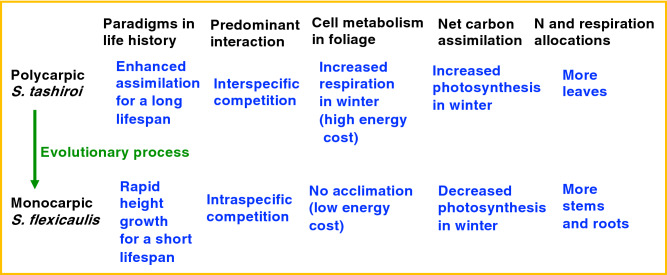
Link between the “height growth” and “assimilation” paradigms in life-history strategies with leaf respiration and nitrogen (N) regulation. Physiological thermal acclimation in *Strobilanthes* leaves accompanied by the evolution from the polycarpic, perennial flowering behavior to the monocarpic, mass-flowering behavior. See the text for more details.

For understory plants in stable overwintering evergreen forests, thermal acclimation is the primary factor for determining carbon assimilation, because the seasonal variations in light are relatively small, and prolonged drought is rare. Polycarpic *S. tashiroi*, having a long-lived lifespan, exhibits winter thermal acclimation, accompanied by a resource allocation bias; its respiration and N allocations are biased toward photosynthetic tissues. In contrast, the monocarpic *S. flexicaulis,* which requires rapid height growth for 6 years after germination, has lost its winter thermal acclimation ability. In this species, respiration and N allocation are biased toward nonphotosynthetic tissues*.* These differences between the two species are represented by the “assimilation” paradigm in *S. tashiroi* and the “height growth” paradigm in *S. flexicaulis* in life-history strategies (Fig. 5). The lack of thermal acclimation in *S. flexicaulis* leaves will be well adjusted to their unique evolution towards the monocarpic, masting behavior within the local *Strobilanthes* group. Taking into account the huge energy costs for thermal acclimation and the required rapid height growth under intensive intraspecific competition, the costly thermal acclimation of foliar cells will decrease the instantaneous height-growth rates and resultantly reduce the plant size and seed production in *S. flexicaulis* because of the shading by the other individual plants. Therefore, the monocarpic *S. flexicaulis* would be unable to adopt the costly thermal acclimation that involves cell metabolism and N reallocation within the lamina, especially under relatively shaded understory environments. The energy costs of protein turnover amount to approximately 16–61% of ATP production rates in *Alocasia* and *Phaseolus* plants^27,28^. Active growth of plant tissues requires large amounts of ATP and NADPH for the construction respiration, which possibly modifies the temperature sensitivity of respiration^21,29^. These facts reveal a previously unknown trade-off related to the regulation of respiration between rapid height growth for a short lifespan (i.e., height growth paradigm) and seasonal physiological acclimation over a long lifespan (i.e., assimilation paradigm).

This finding is parallel to the previously well-known trade-offs related to leaf lifespan at the individual leaf level. Among plant species, long-lived, thick leaves must pay large N, C, and energy costs to ensure that long leaf lifespan^30–32^, but leaf mechanical resistance is not directly correlated with photosynthetic capacity^33^. Furthermore, a shade-to-sun and sun-to-shade transfer experiment in potted seedlings showed that the acclimation in photosynthetic capacity under altered light intensity was smaller in a pioneer tree with a short leaf lifespan than in late-successional trees with long leaf lifespans^34^. Thus, the newly proposed trade-off at the individual plant level is analogous to the well-known trade-offs at the individual leaf level. In this study, we try to provide a link between the “height growth” (for the monocarpic *S. flexicaulis*) and “assimilation” (for the polycarpic *S. tashiroi*) paradigms within life-history strategies with physiological acclimation capacity related to plant lifespan, involving interspecific variations in resource and respiration allocations within a plant body. The monocarpic *S. flexicaulis* has long stems and a high stem mass ratio, while the polycarpic *S. tashiroi* effectively allocates C and N resources and respiration costs into leaves (Fig. 5); the mean stem heights were 113.5 cm and 54.3 cm in the *S. flexicaulis* and *S. tashiroi* plants examined in this study, respectively (data not shown). The low stem respiration rates in *S. flexicaulis* (Fig. 3; Supplementary Table 2) were due to the lignification of long stems to maintain their large overall plant size.

In the polycarpic *S. tashiroi*, the enhanced or maintained carbon gains during cold winters are important for maintaining the long lifespan of the forest understory. This is because carbon starvation within the plant body directly induces plant/tissue death^35–39^. The seasonal variations in photosynthetic and respiration rates were unable to be explained by those in N concentrations in the polycarpic *S. tashiroi* leaves (Fig. 3g–i). However, the enhanced cell metabolism and PNUE in winter would contribute to substantial carbon gains under cold air temperature and ensure their long lifespan of this species, even securing the huge energy costs for thermal acclimation. Seasonal acclimation, which increases the contents of metabolic protein, Rubisco, photoprotective pigments, and osmoles within single leaves in winter, has been reported in many long-lived, overwintering evergreen trees^40–45^. Recent studies using *Arabidopsis* have clarified that plant acclimation to lowered temperatures requires the expression of many genes and is a complex process with multiple steps^43–45^. The polycarpic *S. tashiroi* leaves, but not those of *S. flexicaulis,* exhibited a significant increase in photosynthetic capacity and metabolic respiration in winter. Nevertheless, the molecular mechanisms underlying the lack of acclimation in *S. flexicaulis* are not yet known.

Thermal acclimation of the dark respiration rates of foliar cells has been reported in many vascular plant species in long-term experiments^6,10,46^. Thermal acclimation in the respiratory metabolism of cells depends not only on plant species but also on tissue/organ types^47–49^. The results for *S. tashiroi* also demonstrated that seasonally thermal acclimation is dependent on tissue type; acclimation is greater in photosynthetic tissues than in nonphotosynthetic tissues. However, the opposite results were also reported in a short-term experiment. A 1-week experiment showed that thermal acclimation is less notable in photosynthetic tissues than in nonphotosynthetic tissues in some plant species^49^. This contradiction may be because we have used field-grown understory plants, which have evolutionarily adapted to seasonal temperature variations over the long term. When incorporating the effects of altered temperatures on plant acclimation into climate-warming models, this contradiction may become crucial for predicting global carbon cycling. A meta-analysis clarified that the thermal acclimation of leaf respiration increases with increasing the duration of experimental treatments^17^. Thus, there may still be a gap between the results of laboratory experiments and those of field observations.

We hypothesize that the thermal acclimation capacity has been lost during the evolutional process from the long-lived polycarpic behavior in *S. tashiroi* to the periodic monocarpic mass flowering behavior in *S. flexicaulis,* accompanied with the life-history change from the “assimilation” to the “height growth” paradigms. This study is the first report to analyze physiological acclimation between closely related plants with distinct life-history strategies, together with the known evolutionary history based on molecular evidence. The lack of thermal acclimation in monocarpic *S. flexicaulis* would be associated with a disadvantage under climate warming, because of the lack of homeostatic regulation. The current results suggest that rare understory plants evolving a fast-growing life history are highly vulnerable to global warming, because of their low migration capacity. Approximately 400 *Strobilanthes* species are distributed in the understory of tropical and subtropical forests in the world^50,51^, including many species assessed as VU (vulnerable), CR (critically endangered) and EN (endangered) in the IUCN red list category. Many of these species are also reported to exhibit periodic mass-flowering behavior; for example, *S. flexicaulis,* with masting every 6 years in Japan, *S. fragrans* J.R.I. Wood, with masting every 6 years in Thailand^52^, *S. cerna* Bl. with masting every 9 years in Indonesia^53^, and *S. kunthiana* (Wall. Ex Nees) T. Anders ex Benth. with masting every 12 years in India^54^. Moreover, many *Strobilanthes* species are polycarpic perennials with non-masting behavior, such as *S. tashiroi* and *S. cusia* (Nees) Kuntze in Japan. However, molecular phylogenetic analysis has not yet been completed, and information on flowering behavior is also largely lacking for these species^23^. Further detailed study is greatly needed to elucidate the causal mechanisms of the unique periodic monocarpic behavior and to clarify the linkage among life strategy, physiological acclimation, and the molecular mechanisms underlying the thermal acclimation response in plants. The genus *Strobilanthes* could be a sound model taxon for linking several disciplines in plant biology and for contributing to biodiversity conservation under global warming, especially for rare and endangered species.

## Conclusions

Our results indicate that the seasonal thermal acclimation in cell metabolism (respiration rates measured at a given temperature) is involved in evolution of life history. The seasonal thermal acclimation has been lost during the evolutional process from the polycarpic behavior of *S. tashiroi* to the monocarpic 6-year masting behavior of *S. flexicaulis* within the local area.

Thermal acclimation is the important adaptation for seasonal temperature variations for many plants, involving high energy costs. As in many other plants the polycarpic *S. tashiroi*, with a long lifespan, maintains effective carbon assimilation in the whole plants over seasons with altered air temperatures by thermal acclimation. In contrast, the monocarpic *S. flexicaulis* requires fast height growth during a short growing period (6 years) to avoid shading under high intraspecific competition. If the individual plants suffer from shading by neighboring plants, the carbon gain will largely decrease and resultantly their plant size and seed production will be extensively suppressed (i.e., reduced fitness). We hypothesize that the monocarpic *S. flexicaulis* has lost costly thermal acclimation under the severe height growth competition along with the evolution of life history change.

The representation of linkage between physiological acclimation and the evolution of life history strategy is still rare. More study is urgently needed to clarify this linkage because the rare understory plants with low thermal acclimation and low migration capacity may be vulnerable to global warming.

## Methods

All the plant experiments were carried out in accordance with relevant guidelines. The plant materials used in this study were identified by Dr. Satoshi Kakishima in National Museum of Nature and Sciences.

### Study sites

The study sites were located in the subtropical forests on Okinawa Island on the Ryukyu Islands of Japan. The population of *Strobilanthes flexicaulis* Hayata (26° 38′ N, 127° 55′ E, 280 m a.s.l.) and the population of *Strobilanthes tashiroi* Hayata (26° 48′ N, 128° 16′ E, 365 m a.s.l.) were located on Mt. Yae-dake and Mt. Nishimei-dake, respectively. The *S. tashiroi* population was located at approximately 39 km northeast of the *S. flexicaulis* population. *S. flexicaulis* is a monocarpic, perennial shrub and has the periodic mass flowering of every 6 years, whereas *S. tashiroi* is a polycarpic perennial herb and has a non-masting behavior^22^. Almost all *S. flexicaulis* plants flowered in autumn and winter of 2015, and the seed germination and seedling growth started in spring 2016. The seedling density under the parent plants was 780 ± 324 m^−2^ (mean ± 1 S.D.) in June 2016, showing high intraspecific competition following germination in the monocarpic *S. flexicaulis*. Their seeds do not have intrinsic dormancy after dropping to the ground. In *S. flexicaulis*, all plants examined in summer in 2019 and in winter in 2020 were thus 3 years old.

### Plant materials

Phylogenetic analysis based on the chloroplast and nuclear DNA sequences of the small *Strobilanthes* group within the Ryukyu and Taiwan Islands, has been published. According to the results, *S. flexicaulis* and *S. tashiroi* are closely related species. Furthermore, their evolutionary processes have been estimated as follows: polycarpic perennial behavior via monocarpic perennial behavior, to monocarpic mass/synchronous flowering behavior within the local area^23^. Therefore, it is predicted that the periodical behavior of *S. flexicaulis* has locally evolved from the polycarpic behavior of *S. tashiroi*^23^.

### Climatological measurements

The air temperature at approximately 1 m high was measured at every 1 h. with a thermistor sensor (TER-51i, T&D Co Ltd, Nagano, Japan) at just near the *S. flexicaulis* population from 2017 to 2020. From 2017 to 2020, the mean annual precipitation was 2415 mm at the closest climatological station in Nago (observation by the Japan Meteorological Agency). No snow accumulation was found in the understory.

### Photosynthetic measurements

The photosynthetic light-response curves and water–vapor stomatal conductance were measured in the fully expanded young leaves on Sep. 23 and Sep. 24 (summer) in 2019 and on Jan. 24 and 25 (winter) in 2020 with a portable open gas exchange system (LI-6400; LI-COR Inc., Lincoln, NE, USA) in the field. The measurements were conducted with 400 µmol mol^−1^ CO_2_ in the inlet gas stream. The exposed photon flux density (PFD) was stepwise decreased from 1500, 1000, 700, 400, 300, 200, 100, 70, 50, 30, 20, 10, 7, 4 and 0 µmol m^−2^ s^−1^ with red-blue light emitting diodes. The relative humidity (RH) in the outlet gas stream was adjusted to the ambient air RH. All leaf gas exchange measurements were conducted before noon to avoid the midday stomatal closure. In each season, in each species we selected 7–8 individual plants which the canopy leaves did not suffer from damage by herbivores and the top canopies were not shaded by the other plants. The fully expanded young leaves of individual plants from each species were used for the measurements.

The initial slope (*Φ*), the light compensation point (LCP) and dark respiration rates (*R*_d_) were calculated for each leaf from the linear regression between low PFDs (10, 7, 4 and 0 µmol m^−2^ s^−1^) and the corresponding net assimilation rates (*A*_n_) (*r*^2^ = 0.999–0.858). Light-response curves in net assimilation rates (*A*_n_) in Supplementary Fig. 2 were calculated by using the following equation:

$$A_{n} = \frac{{\Phi \;I + A_{{max}} - \sqrt {\left( {\Phi I + A_{{max}} } \right)^{2} - 4\;\theta \;\Phi \;I\;A_{{max}} } }}{{2~\theta }} - R_{d}$$

where *A*_max_ are the maximum gross assimilation rates, *I* is the PFD, and *θ* is the curvature. When *θ* = 0, the fitting curve is converted into rectangular hyperbola equations; when *θ* = 1, it is converted into Blackman equations. The values of *θ* in *S. flexicaulis* were 0.805 and 0.678 in winter and summer, respectively; those in *S. tashiroi* were 0.669 and 0.701 in winter and summer, respectively.

### Dark respiration measurements

After the photosynthetic measurements, we collected seven individual plants for which the top canopies were not directly covered by the other plants, in each season or each species. Their stem heights were 113.5 ± 9.0 and 54.3 ± 16.9 cm (mean ± 1 S.D.) for *S. flexicaulis* and *S. tashiroi*, respectively. Their stem diameters near the ground were 9.0 ± 2.0 and 4.8 ± 1.1 mm for the monocarpic *S. flexicaulis* and the polycarpic *S. tashiroi*, respectively.

We separated the individual plants into three parts (leaves, stems, and roots) with scissors, and immediately measured their dark respiration rates under the field conditions. The plant parts were put into a closed plastic box with a small fan (1.3 L, 5 L or 14.5 L in volume; the size of the plastic box was selected according to the volume or length of the samples), and then the box was covered with black cloth. To measure the air temperature, copper-constantan thermocouples were placed in the box together with the plant parts. Here, we assumed that the plant temperature was in equilibrium with the air temperature in the box. We measured the increasing rates in air CO_2_ concentration in the box for approximately 3–5 min, using a nondispersive infrared CO_2_ gas analyzer (GMP343, Vaisala Inc., Helsinki, Finland) connected with a data logger (GL240, Graphtec Co. Ltd., Yokohama, Japan). From the CO_2_ increasing rates and the box volume, we calculated the dark respiration rates. After the field measurements, the plant samples were dried at 60 °C for at least 3 days and then the dry mass was weighed. Dry mass-based respiration rates were calculated. Furthermore, to evaluate metabolic activity in cells at a given temperature, the respiration rates were standardized at 19 °C and 28 °C in both seasons by using Q_10_ = 2^6,19^. The chosen values of 19 °C and 28 °C were the mean box temperatures in winter and summer, respectively.

### Allocations of dry matter, whole-plant respiration and N in plants

To evaluate the interspecific variations in plant form, the plant samples used for respiration measurements were used to calculate dry matter allocation. Leaf areas of individual plants were calculated from the leaf dry mass in individual plants and the mean values of leaf mass per area (LMA) in each species. The standardized whole-plant respiration rates were calculated from the mean dry matter allocation in each species and the mean dark respiration rates of each tissue in each season.

The N concentrations in the plant tissues were also examined. Each tissue in individual plants was milled into powder with a cutter mill (ABSOLUTE 3, Osaka Chemical Co. Ltd., Osaka, Japan). The N concentrations were measured with an NC analyzer (Vario Max CN, Elementar Analysensysteme GmbH Co. Ltd., Hanau, Germany). The standardized N and respiration allocations among tissues in the plants were calculated from the mean dry matter allocation in each species and the mean values of N and respiration in each tissue and each season.

### Statistics

Statistical analyses were conducted with R Ver. 3.0.2 (R Development Core Team, R Foundation for Statistical Computing, Vienna, Austria). To evaluate seasonal acclimation, the significant differences between winter and summer were examined with ANOVA at *P* < 0.05 (Supplementary Table 1). The significant differences between species were also examined with ANOVA at *P* < 0.05 (Supplementary Table 2). The mean (1 S.D.) values and all statistical results are shown in the “Supplementary materials”.

## Supplementary Information

Supplementary Information.
