# Supplementary material for: Evolutionary loss of thermal acclimation accompanied by periodic monocarpic mass flowering in Strobilanthes flexicaulis
Source: Sci Rep. 2021 Jul 12;11:14273. doi: 10.1038/s41598-021-93833-1 (PMC8275617; doi:10.1038/s41598-021-93833-1)
Supplement: Supplementary file 1 — Supplementary Information. [file 41598_2021_93833_MOESM1_ESM.pdf]

Evolutionary loss of thermal acclimation accompanied by periodic monocarpic mass flowering in *Strobilanthes flexicaulis*

Atsushi Ishida, Tomomi Nakamura, Shin-Taro Saiki, Jin Yoshimura, Satoshi Kakishima

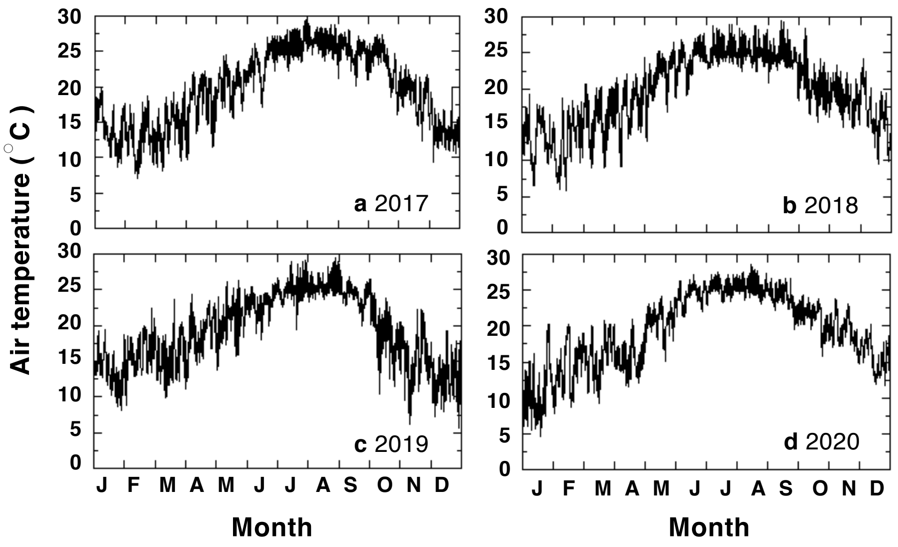

Supplementary Figure 1. The seasonal variations in air temperature from 2017 to 2020 in the field. (**a**) 2017, (**b**) 2018, (**c**) 2019 and (**d**) 2020.

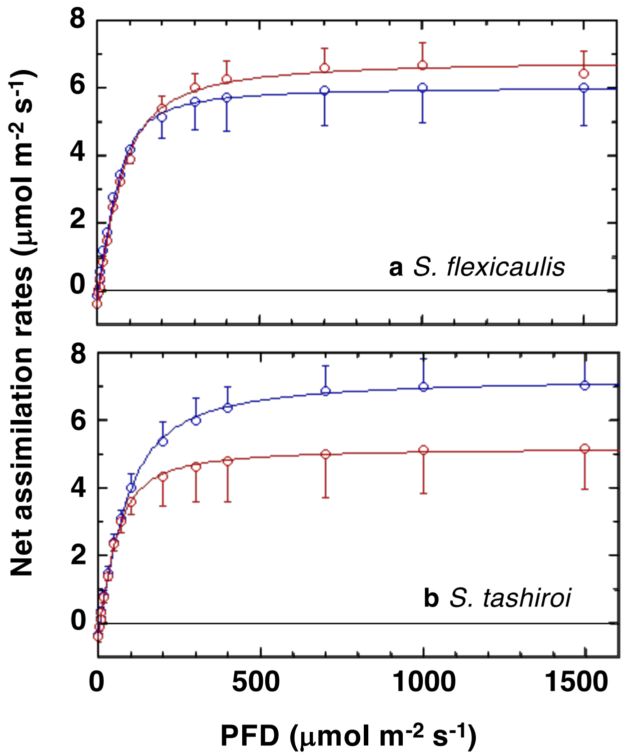

Supplementary Figure 2. Photosynthetically light-response curves in (**a**) the monocarpic *Strobilanthes flexicaulis* and (**b**) the polycarpic *S. tashiroi* in winter (blue) and summer (red). PFD is photon flux density. The leaf gas exchange was measured in the field setting. Bars show + 1 S.D.

Supplementary Table 1. The mean values (1 S.D.) and the statistical results in one-way ANOVA between seasons in each species. Bolds show significant differences ($P < 0.05$).

Plant traits	Comparison	Species	Winter	Summer	*F*-value	*P*-value
Area-based photosynthesis						
Max. net assimilation rate	Seasons	*S. flexicaulis*	6.07 (1.12)	6.70 (0.63)	1.68	0.219
(μmol m-2 s-1)	**Seasons**	***S. tashiroi***	**7.11 (0.91)**	**5.45 (1.30)**	**7.96**	**0.014**
Max. stomatal conductance	Seasons	*S. flexicaulis*	0.140 (0.082)	0.203 (0.022)	3.85	0.073
(mol m-2 s-1)	Seasons	*S. tashiroi*	0.215 (0.058)	0.221 (0.052)	0.045	0.836
Light compensation point	**Seasons**	***S. flexicaulis***	**2.3 (0.74)**	**6.0 (1.88)**	**16.3**	**0.002**
(μmol m-2 s-1)	Seasons	*S. tashiroi*	5.8 (1.90)	6.1 (1.50)	0.117	0.738
Initial slope of light response	Seasons	*S. flexicaulis*	0.064 (0.012)	0.067 (0.014)	0.185	0.675
	Seasons	*S. tashiroi*	0.061 (0.011)	0.069 (0.010)	2.18	0.164
Dark respiration rate	**Seasons**	***S. flexicaulis***	**0.180 (0.034)**	**0.396 (0.101)**	**28.8**	**< 0.001**
(nmol g-1 s-1)	Seasons	*S. tashiroi*	0.356 (0.124)	0.448 (0.142)	0.00008	0.978
A max/*R* d ratio	**Seasons**	***S. flexicaulis***	**34.7 (8.14)**	**17.9 (4.98)**	**21.7**	**< 0.001**
	**Seasons**	***S. tashiroi***	**22.5 (8.48)**	**13.8 (3.20)**	**7.30**	**0.018**
Measured leaf temperature	**Seasons**	***S. flexicaulis***	**20.7 (0.4)**	**28.9 (0.5)**	**1157**	**< 0.001**
(°C)	**Seasons**	***S. tashiroi***	**23.8 (0.3)**	**28.5 (0.4)**	**580**	**< 0.001**
Mass-based dark respiration (nmol g-1 s-1)						
Leaf at 19°C	Seasons	*S. flexicaulis*	2.91 (0.38)	2.90 (0.81)	0.0009	0.976
	**Seasons**	***S. tashiroi***	**5.04 (1.27)**	**2.90 (1.59)**	**16.3**	**< 0.001**
Stem at 19°C	Seasons	*S. flexicaulis*	0.77 (0.20)	0.74 (0.17)	0.121	0.734
	**Seasons**	***S. tashiroi***	**1.32 (0.15)**	**1.12 (0.15)**	**6.02**	**0.030**
Root at 19°C	Seasons	*S. flexicaulis*	3.37 (0.52)	3.45 (0.56)	0.0774	0.786
	Seasons	*S. tashiroi*	**2.82 (1.82)**	**3.65 (1.18)**	1.036	0.329
Leaf at 28°C	Seasons	*S. flexicaulis*	5.43 (0.71)	5.41 (1.51)	0.0009	0.976
	**Seasons**	***S. tashiroi***	**9.41 (2.38)**	**5.42 (1.09)**	**16.3**	**< 0.001**
Stem at 28°C	Seasons	*S. flexicaulis*	1.44 (0.37)	1.38 (0.32)	0.121	0.734
	**Seasons**	***S. tashiroi***	**2.46 (0.28)**	**2.09 (0.28)**	**6.03**	**0.030**
Root at 28°C	Seasons	*S. flexicaulis*	6.29 (0.97)	6.44 (1.05)	0.0775	0.786
	Seasons	*S. tashiroi*	5.26 (3.39)	6.81 (2.20)	0.674	0.428
N concentrations (mmol N g-1)						
Leaf	Seasons	*S. flexicaulis*	2.34 (0.07)	2.47 (0.16)	3.70	0.078
	Seasons	*S. tashiroi*	1.87 (0.20)	1.69 (0.12)	3.78	0.076
Stem	Seasons	*S. flexicaulis*	0.83 (0.09)	0.79 (0.07)	1.95	0.189
	**Seasons**	***S. tashiroi***	**0.75 (0.11)**	**0.96 (0.11)**	**12.7**	**0.004**
Root	Seasons	*S. flexicaulis*	0.97 (0.15)	0.97 (0.18)	0	0..995
	**Seasons**	***S. tashiroi***	**0.69 (0.08)**	**0.91 (0.14)**	**12.5**	**0.004**

Supplementary Table 2. The mean values (1 S.D.) and the statistical results in one-way ANOVA between species. Bolds show significant differences ($P < 0.05$).

Plant traits	Comparison	*S. flexicaulis*	*S. tashiroi*	*F*-value	*P*-value
Area-based photosynthesis in winter					
Max. net assimilation rate	Species	6.07 (1.12)	7.11 (0.91)	1.337	0.270
Max. stomatal conductance	Species	0.140 (0.082)	0.215 (0.058)	3.846	0.074
**Light compensation point**	Species	2.3 (0.74)	5.8 (1.90)	**13.51**	**0.003**
Initial slope of light response	Species	0.064 (0.012)	0.061 (0.011)	0.0065	0.937
**Dark respiration rate**	**Species**	0.180 (0.034)	0.356 (0.124)	**6.994**	**0.021**
Area-based photosynthesis in summer					
**Max. net assimilation rate**	**Species**	**6.70 (0.63)**	**5.45 (1.30)**	**5.384**	**0.037**
Max. stomatal conductance	Species	0.203 (0.022)	0.221 (0.052)	0.728	0.409
Light compensation point	Species	6.0 (1.88)	6.1 (1.50)	0.0017	0.968
Initial slope of light response	Species	0.067 (0.014)	0.069 (0.010)	0.053	0.821
Dark respiration rate	Species	0.396 (0.101)	0.448 (0.142)	0.648	0.436
Mass-based dark respiration (nmol g-1 s-1)					
**Leaf in winter**	**Species**	**2.91 (0.38)**	**5.04 (1.27)**	**18.0**	**0.0011**
**Stem in winter**	**Species**	**0.77 (0.20)**	**1.32 (0.15)**	**34.2**	**<0.001**
Root in winter	Species	3.37 (0.52)	2.82 (1.82)	0.590	0.457
Leaf in summer	Species	5.41 (1.51)	5.42 (1.09)	0.0002	0.990
**Stem in summer**	**Species**	**1.38 (0.32)**	**2.09 (0.28)**	**20.2**	**<0.001**
Root in summer	Species	6.44 (1.05)	6.81 (2.20)	0.169	0.688
N concentrations (mmol N g-1)					
Leaf in winter	Species	2.34 (0.07)	1.87 (0.20)	34.4	<0.001
Stem in winter	Species	0.83 (0.09)	0.75 (0.11)	2.2	0.162
**Root in winter**	**Species**	**0.97 (0.15)**	**0.69 (0.08)**	**19.0**	**<0.001**
**Leaf in summer**	**Species**	**2.47 (0.16)**	**1.69 (0.12)**	**106**	**<0.001**
**Stem in summer**	**Species**	**0.79 (0.07)**	**0.96 (0.11)**	**11.9**	**0.0048**
Root in summer	Species	0.97 (0.18)	0.91 (0.14)	0.5	0.500
Plant form					
**Leaf area ratio (m2 g-1)**	**Species**	**0.00324 (0.00046)**	**0.00704 (0.00331)**	**18.1**	**<0.001**
**Leaf mass ratio (g g-1)**	**Species**	**0.157 (0.022)**	**0.285 (0.108)**	**18.9**	**<0.001**
**Stem mass ratio (g g-1)**	**Species**	**0.664 (0.034)**	**0.575 (0.140)**	**5.53**	**0.027**
Root mass ratio (g g-1)	Species	0.179 (0.026)	0.140 (0.073)	3.86	0.060
**Leaf mass per area (g m-2)**	**Species**	**48.3 (2.4)**	**37.2 (4.5)**	**47.1**	**<0.001**
